# A dynamic analysis of the relationship between investor sentiment and stock market realized volatility: Evidence from China

**DOI:** 10.1371/journal.pone.0243080

**Published:** 2020-12-04

**Authors:** Yanhui Chen, Hanhui Zhao, Ziyu Li, Jinrong Lu

**Affiliations:** School of Economics and Management, Shanghai Maritime University, Shanghai, China; Hunan University of Science and Technology, CHINA

## Abstract

Investor sentiment is a research focus in behavior finance. This paper chooses five proxy variables according to China’s reality and uses a two-step principal component analysis to construct an investor sentiment index. The five proxy variables are the number of new stock accounts, turnover ratio, margin balance, net active purchasing amount, and investor attention. In the final part of this study, using the price data from the Shanghai and Shenzhen Security Exchange, this paper investigates the dynamic relationship between investor sentiment and stock market realized volatility based on the thermal optimal path. The empirical results show that when the market fluctuates severely, investor sentiment leads stock market realized volatility over one or two steps. The prediction power is also checked. The results indicate that investor sentiment indeed forecasts the realized volatility. This research supports regulators and financial institutions in taking advanced measures.

## Introduction

The stock market has been regarded as a complex system because different types of agents operate within it [1]. While institutional investors have rational expectations, no one person does. With the development of behavioral finance, increasing attention has been given to the influence of investors’ irrational factors on the stock market [2]. Accordingly, investor sentiment is a research focus in this field.

Researches departing from rational agents in financial markets often consider the influence of investor sentiment. For example, De Long et al. [3] indicate that sentiment indices are a market pseudo signal that can be used by professional arbitrageurs. Kenneth and Statman [4] believe that investor sentiment studies are important because they expose the stock market’s biases and exploiting those biases can help investors seize opportunities to earn extra returns. Thus, many academic and industry researchers have explored three key research questions: i.e., the construction of an index that can describe investor sentiment accurately and scientifically, evaluating the impact of investor sentiment on the stock market, and whether investor sentiment should be used in stock market forecasts. In recent years, many researchers have devoted themselves in relevant researches. Debata, Dash [5] examine the impact of investor sentiment on the liquidity of 12 emerging stock markets. Han and Li [6] construct a market-based sentiment index and indicate that sentiment is a reliable momentum predictor with monthly frequency in China. Guo, Sun [7] suggest that the stock market price is not always lead by sentiment data, which can be used to predict the stock price only when the stock has high investor attention. Renault [8] constructs intraday investor sentiment indicators and finds that the first half-hour change in investor sentiment predicts the last half-hour of Standard & Poor’s 500 Index ETF funds return. Kumari and Mahakud [9] probe the influence of investor sentiment on the predictability of Indian stock market volatility. Yu and Yuan [10] find that stock market expected returns are positively related to market conditional variance over low-sentiment periods, but are unrelated to conditional variance over high-sentiment periods.

Generally, investor sentiment can be measured in four ways. Robert Shiller proposed the first method using a questionnaire designed and distributed by his project team, which has been investigating retail and institutional investors continuously since 1989. However, this method doubtlessly requires much time and money, and such a huge project may be out of the financial reach of ordinary researchers. Although the data from their website can be downloaded by other researchers, only two countries (US and Japan) are included in the survey. Economists are suspicious of individual survey data because they believe that a potential gap exists between how people respond to a survey and how they actually behave [11]. Following this line of thinking some researchers use a single index, which can also be obtained from surveys as a proxy for investor sentiment. For example, Schmeling [12] uses consumer confidence as a proxy for individual investor sentiment and examines its effect on expected stock returns internationally. Wang [13] also uses a consumer confidence index to reflect investor sentiment and investigates the impact of investor sentiment on the mean-variance relationship. Ho and Hung [14] use three survey indices: the Conference Board’s Consumer Confidence Index (CCI), the Investors’ Intelligence Survey Index (II), and the University of Michigan Consumer Sentiment Index (MS) to proxy for the investor sentiment indicator. The second method uses a single market indicator as the sentiment indicator. For example, Baker and Stein [15] take market liquidity as a sentiment indicator. Chen, Chen [16] use turnover by volume to proxy investor sentiment. The third method is proposed by Baker and Wurgler [17], who creates an investor sentiment index by exacting principal components from a group of proxy variables. Their practical method is widely used by researchers worldwide. For example, Yu and Yuan [10] use Baker and Wurgler’s index to identify high-sentiment periods. Kumari and Mahakud [9] employ ten indirect measures of sentiment proxies to create an index taking the first principal component of these ten indirect measures and the lagged components of these sentiment variables. Qadan and Nama [18] capture investor sentiment by nine different proxies. The key issue with this method is how to choose proxy variables. Different countries must modify the proxy variable set according to their actual conditions, because the indices published by different countries are not the same and their market rules differ. Following advances in programming, some researchers collect user comment data from financial review websites and obtain investor sentiment through semantic analysis. For example, Kim and Kim [19] use more than 32 million messages on 91 firms posted on the Yahoo! Finance message board to measure investor sentiment. Renault [8] analyzes the messages published on the social media platform StockTwits to construct intraday investor sentiment indicators. Guo et al. [7] collect the user comment data from a popular professional social networking site for the China stock market and then obtains investor sentiment data through semantic analysis. However, there are always some random bystanders who talk nonsense. For example, Internet water armies operate in China. Certain organizations would like to hire these groups of people to guide public opinions so that they may benefit from the impact of public opinions. More importantly, although 99.7% of stock accounts were held by natural persons (retail investors) in China according to the statistical data from China Securities Depository and Clearing Corporation Limited, they only control less than 10% of the market value. The financial review website data are only concerned with retail investor sentiments, which do not represent investor sentiments in general.

We consider the cons and pros of the aforementioned three methods. Baker and Wurgler’s method is still the most effective for measuring investor sentiment. Thus, five proxy variables are chosen according to the real-world situation in China and the availability of data for this paper. We specifically did not use the number of initial public offerings (IPOs) because they are strictly controlled by the China Securities Regulatory Commission (CSRC). As a result, unlike the registration-based IPO system, the timing and the number of IPOs are not decided by the market directly. Moreover, Chinese investors are enthusiastic about new stocks. The value of almost all new stocks rockets during the first day. Thus, we don’t consider the average first-day returns on IPOs either. The closed-end fund discount is also not considered because open-end funds account for nearly 90% of the market share in China and even in the US, its scale grows continuously. In a later study, Baker et al. [20] indicated that data limitations and country-specific sentiment indices should be considered. Han and Li [6] also observe that sentiment proxies are subject to data availability. The five proxy variables that we use are the number of new stock accounts, turnover ratio, margin balance, net active purchase amounts and investor attention. The turnover ratio is the same proxy variable as used in Baker and Wurgler’s research, which suggested that turnover ratio is related to liquidity. In contrast to the US stock market, the Chinese stock market is open to retail investors without any constraints. Individuals can register a stock account just with their identification documents and bank accounts in which there can be no money. Thus, more people tend to open an account when the market is doing well. Margin trading can be long or short, which increases the liquidity of the market as a whole. If the margin balance increases, investors are optimistic about the future and vice versa. The net active purchase amount represents the investors’ positivity in the stock market. If the investors turn bullish, they will throw more funds into the market and buy more shares. For many years, investor attention has been recognized as a factor associated with stock prices and liquidity [21]. After the seminal work of Da et al. [22], who used the Google Search Volume Index (GSVI) to measure investor attention, a lot of studies suggested that the search volume index is indicative of stock market returns and volatility, such as Xu et al. [23] and Swamy et al. [24]. In this paper, we consider using the Baidu Search Volume Index, which is developed by the largest Internet search engine in China, to represent investor attention. The lead-lag relationship is also considered in this research; therefore, both the proxy variables and their one-lag values are included. To obtain more information from the proxy variables, we use principal component analysis (PCA) twice. The first PCA is based on the proxy variables and their one-lag values. We choose four indictors from the first PCA according to the indicators’ correlation coefficients with the first component. We then use PCA again to obtain the investor sentiment.

With the globalization of financial market interactions, more features of complex systems emerge in the stock market, such as its nonlinear, dynamic, and self-organizing qualities. Thus, traditional linear and parametric econometric models are not suitable for studying this complex system. Researchers increasingly turn to the complicated futures of financial markets. Dergiades [25] explores the nonlinear causal linkage between investors’ sentiment dynamics and stock returns for the US economy. Kumari and Mahakud [9] employ the nonlinear conditional GARCH class of models in their analysis. Nam and Seong [26] combine complex system methodology with machine learning in financial news-based stock movement prediction. Few studies in the literature have explored the time-varying characteristics of the relationship between investor sentiment and stock market realized volatility in a complex system; therefore, some time-varying models were employed to study this issue. In the final part of this research, we investigate the relationship between investor sentiment and stock market volatility based on the thermal optimal path (TOP) method, which was proposed to identify and quantify the time-varying lead-lag structure between two time series.

The contributions of this research are twofold. First, we construct an investor sentiment index considering investor attention, which is represented by the Baidu Search Volume Index. The results indicate that this sentiment index reduces the total variation and improves forecasting accuracy when it is used to forecast realized volatility. Second, we explore the dynamic relationship between the investor sentiment index and realized volatility. The results indicate that investor sentiment does not always lead the realized volatility. When the market is volatile and the stock index climbs to a high level, investor sentiment leads the realized volatility. However, when the market is flat (the volatility is low), the investor sentiment lagged the realized volatility. Our findings will help the government and related institutions to avoid financial risk in China.

**Fig 1:**
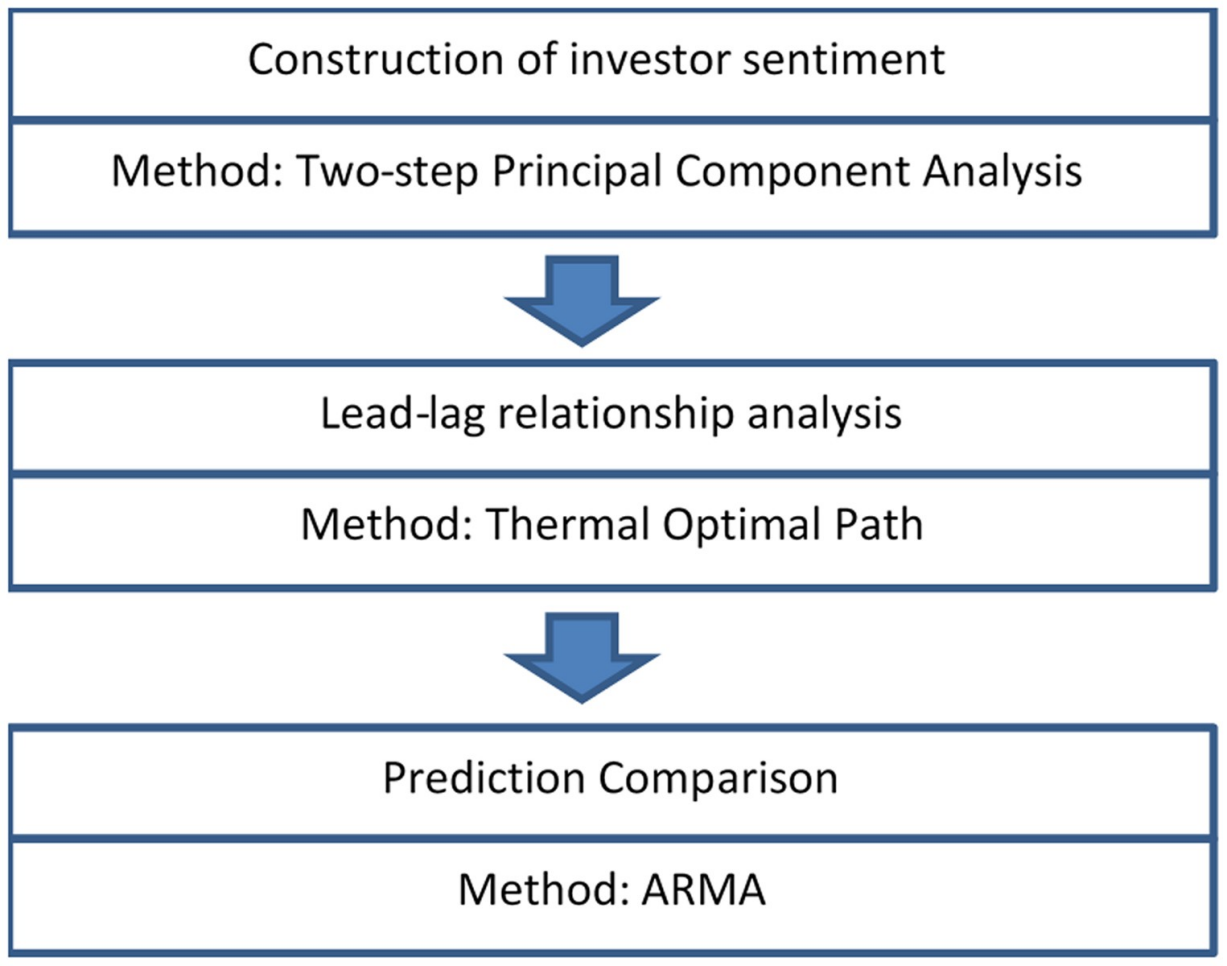
Flow chart for this research.

The structure of this paper is as follows: in Section 2, we introduce the proxy variables and the procedure of constructing an investor sentiment index by a two-step PCA. Section 3 presents the dynamic lead-lag relationship between investor sentiment and realized volatility using the TOP method. The prediction performance of the investor sentiment is also discussed in this part. Section 4 summarizes the contents of this paper and briefly introduces our future work briefly. A flow chart for this research is presented in Fig 1.

## Construction of investor sentiment indicator

### Proxy variables

The selection of proxy variables is the most disputed topic in constructing an investor sentiment index. Although different countries publish similar statistical data for their financial markets, different financial markets operate and are managed in different ways. As a result, different proxy variables should be considered in building an investor sentiment index. After a comprehensive consideration and analysis, this paper chose more reasonable and realistic proxy variables according to the real-world situation of the Chinese stock market. The proxy variables considered in this paper include the number of new stock accounts, margin balance, turnover ratio, net active purchase amount, and investor attention, as represented by the Baidu Search Volume Index. The first four proxy variables were downloaded from Wind, a financial data and analysis tool service provider in mainland China. The Baidu Search Volume Index was obtained from the Data Share Home website (http://www.datasharehome.com), which provides data crawling services at a cost. The details for each proxy variable are as follows.

#### Number of new stock accounts (INR)

The number of new stock accounts per month represents the mood of the potential investors in the Chinese stock market. If the stock market is expected to climb, potential investors will rush to register accounts and participate in the market with high trading enthusiasm. But if the market is flat or bearish, existing investors are pessimistic, speculative demand will weaken, and few potential investors are willing to open accounts. In other words, the number of new investors will decline. There is little change in the number of new stock accounts in the European and American markets every month because these markets are relatively mature and the structure of their investors is reasonable, i.e., most investors are institutional. Therefore, few foreign scholars chose the number of new investors as a proxy variable for investor sentiment. However, the Chinese financial market is far from perfect, the number of retail investors is large, and the changes in the number of new stock accounts can reflect whether potential investors are optimistic about the later stage of the stock market.

#### Margin balance (SMT)

Margin trading is a common trading model for mature stock. Margin trading can be long or short, which increases the overall liquidity of the market. When the margin balance increases, investors are optimistic, the market investment atmosphere is good, and the investors’ enthusiasm for going long is high. In contrast, a reduction in margin balance indicates that investors are inclined to sell their shares, the market investment atmosphere is pessimistic, and the investors are in a wait-and-see mood. The margin balance was considered in analyzing stock market returns; e.g., Zhang, Seyedian [27] observe a strong market momentum effect and a significant causal relation between prior stock returns and margin balance. Therefore, this paper uses the sum of Shanghai and Shenzhen stock exchanges” margin balances as one proxy variable.

#### Turnover ratio (TURN)

The turnover ratio is similar to the turnover in volume, which also reflects the overall activity and liquidity of the securities market. The higher the turnover ratio is, the higher the investor sentiment, and the more frequently the market transacts, i.e., people actively buy and sell stocks actively [5]. Many researchers have used turnover ratio to proxy market liquidity, such as Jun, Marathe [28] and Dey [29]. Dey [29] also observes that investors expect higher returns from high-turnover markets. Turnover ratio as an indicator of investors’ transaction behavior, can reflect the investors’ opinion on future market returns. According to the technical analysis theory, turnover ratio is a leading indicator of price change. If the turnover ratio is too low or too high, it may suggest that the market is oversold or overbought. Therefore, this paper chooses the average turnover ratio in both the Shanghai and Shenzhen stock exchanges as one of the proxy variables. Specifically, this paper uses the ratio of the total monthly trading volume to the total shares listed on the Shanghai and Shenzhen stock exchanges as the average turnover ratio.

#### Net active purchase amount (FUND)

The net active purchase amount can be regarded as a sign of the operation of investors’ capital and as an important factor that can affect the short-term trends in the stock market. The difference between the main capital inflow and outflow is the net active purchase amount on that day. The size of the net active purchase amount determines the strength of capital driving stock prices up and down, and supposedly has a positive correlation with stock prices supposedly. The net active purchase amount reflects informed investors’, usually institutional investors’, optimistic or pessimistic sentiment about certain sectors or individual stocks. The net active purchase amount not only represents the informed investors’ sentiment, but also stimulates retail investors’ sentiment. In the Chinese stock market, retail investors account for more than 80% of all investors, but at the same time, their proportion of funds is less than 10%, while the amount of institutional funds is abundant. This situation has caused a "herding effect"; i.e., retail investors prefer to trade with the main funds, which is commonly known as "following the banker". If the net active purchase amount is positive, retail investors believe that the sector or stock will be the main force to lift stocks in the future. Retail investors are in high spirits and actively follow this trend. In contrast, if the active purchase amount is negative, they will panic-sell.

#### Investor attention (SVI)

With the increasing popularity of networks, the public tend to use search engines to collect information. In China, retail investors constitute a majority of the stock market investors. When the market is bearish, they hold their money and are more interested in low-risk investment. When the market begins to soar, the public check on market information using a search engine. Some people with a stock market account will save money in their accounts to buy mutual funds and stocks, while other people without a stock market account will register new accounts and start their investment career. As Da et al. [22] indicate, searching for information about stock markets can be used as a measure of attention: i.e., if you search for the stock index information, you are undoubtedly paying attention to the stock market. As Baidu accounted for 76.42% of the search engine market share in China from July 2018 to July 2019 and has published search volume indices for various keywords since January 2011. This paper uses a search volume index crawled from Baidu to proxy for investor attention.

**Fig 2:**
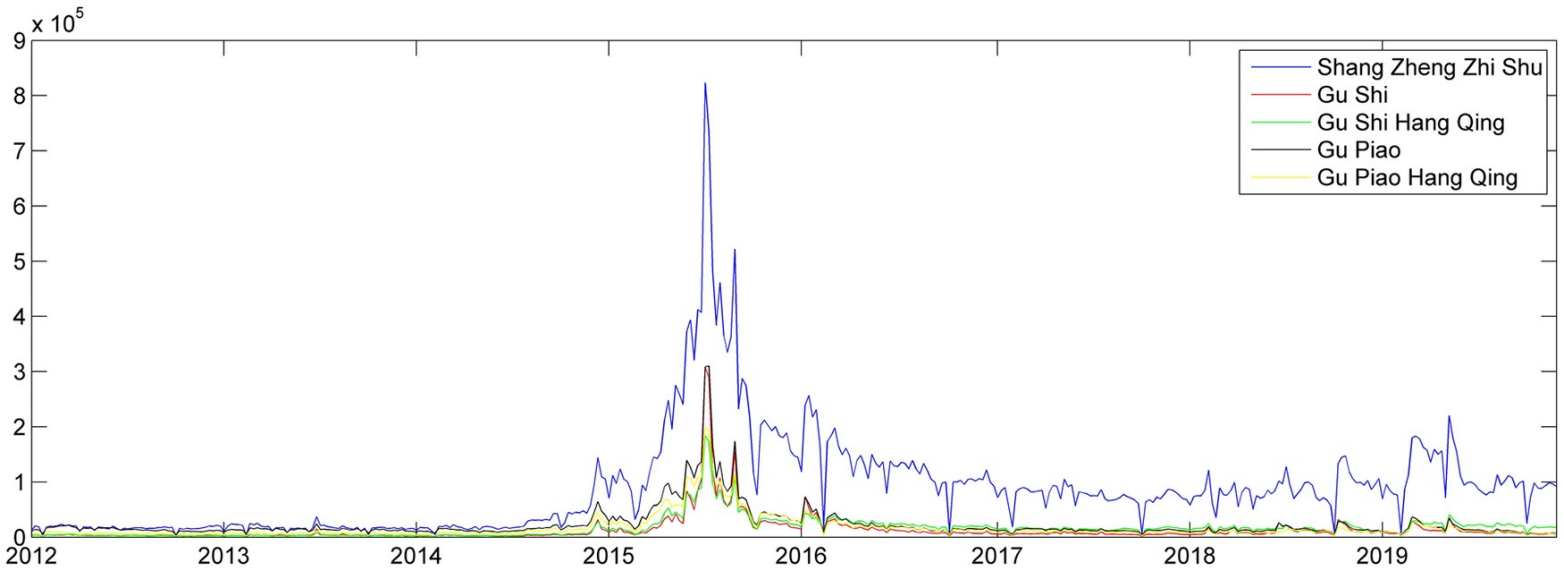
Search volume index of the potential keywords (from April 1st, 2012 to November 30^th^, 2019)^a^. ^a^The data was downloaded from http://index.baidu.com.

Usually investors use the name of the stock index as the keyword to find the market information. Thus, this research chooses search keywords based on quotations of the stock index in financial news. Financial websites use abbreviations for the stock indices and indicate their full names under the abbreviations. We also choose some keywords used by financial website to report the stock market, i.e., “Gu Shi Hang Qing”. Thus, we choose 13 potential keywords (see S1 Table). According to their daily average search volumes, we use “Shang Zheng Zhi Shu”, “Gu Shi”, “Gu Shi Hang Qing”, “Gu Piao”, and “Gu Piao Hang Qing”. The time series of search volumes of these five keywords are presented in Fig 2. The search volumes of “Shang Zheng Zhi Shu” show a similar trend to the other four keywords. As the Shanghai stock market was the first stock market in China after 1949, the listed companies are state-owned enterprises or enterprises with capital more than 100 million yuan. According to the statistic report in the end of 2019, the circulation market value in the Shanghai Stock Exchange(SSE) is about 100 trillion more than that in the Shenzhen Stock Exchange(SZSE). The stock index trends in the SSE reflect the economic situation in China. Although the formal and most-cited stock index in the SSE is its Composite Index (SSECI)(000001.ss), the public tend to use the stock index of the SSE (or “Shang Zheng Zhi Shu” in Chinese phonetic alphabet) as the keyword to search the stock market information.

### Two-step PCA

This paper uses a two-step PCA to construct an investor sentiment index. PCA is a method for reducing dimensions. Basic PCA is popular in econometrics and statistics. Although PCA reduces the number of analysis variables, it minimizes the loss of information contained in the original variables; hence, fewer comprehensive indicators are used to reflect the main information for multiple-related variables. The details for the basic PCA used in this paper, can be found in [30, 31]. In this paper, we cannot directly conduct PCA using the original data for the five proxy variables directly because of large differences in the units of measure. Thus, we must first standardize the original data, which increases the comparability among the proxy variables, and then use PCA in two steps to obtain the investor sentiment.

These five proxy variables (INR, SMT, TURN, FUND, and SVI) may lead investor sentiment trends, thus, we introduce their first order lag terms in the PCA. Specifically, the five proxy variables and their first order lag terms constitute analysis variable set. Margin trading began in 2010 and the earliest data included in WIND are the monthly data from April 2012. In the very beginning, margin trading soared and increased monotonically. Thus, we wash out the data from 2012 and our study period is from January 2013 to November 2019, when we started this research. The margin balance and net active purchase amount are measured with nominal prices, which will be impacted by the economy growth. Therefore, we transfer it into real value using the Consumer Price Index. The correlation between the proxy variables is analyzed. Table 2 indicates that all the proxy variables are correlated with each other.

However, not all of the proxy variables are correlated with the other four variables. For example, the correlation coefficients between INR_t_ and SMT_t_ / SMT_t-1_ / TURN_t-1_ are larger than 0.5, which indicates moderately strong correlation [32], but the coefficients between INR_t_ and FUND_t-1_ / SVI_t-1_ are smaller than 0.25 [32], which indicates weak correlation. Table 3 shows the correlation coefficients between the realized volatility and the proxy variables. The results indicate that both the net active purchase amount (FUND_t_) and its first-order lag term correlate with the realized volatility weakly. Thus, we abandon it in the following parts. Tables 1 and 2 also indicate that it is necessary to use PCA to concentrate the information implied in the proxy variables so that investor sentiment can be abstracted from the proxy variables.

**Table 1:** The correlation coefficients between proxy variables.

	INR_t_	SMT_t_	TURN_t_	FUND_t_	SVI_t_	INR_t-1_	SMT_t-1_	TURN_t-1_	FUND_t-1_	SVI_t-1_
INR_t_	1.0000									
SMT_t_	0.6714	1.0000								
TURN_t_	0.4743	0.6719	1.0000							
FUND_t_	0.3321	0.5900	0.3104	1.0000						
SVI_t_	0.4264	0.7030	0.6781	0.5650	1.0000					
INR_t-1_	0.4847	0.7006	0.6000	0.2437	0.5531	1.0000				
SMT_t-1_	0.5475	0.9466	0.6271	0.6212	0.8393	0.6713	1.0000			
TURN_t-1_	0.5004	0.6163	0.6642	0.5761	0.8050	0.4741	0.6708	1.0000		
FUND_t-1_	0.1233	0.4936	0.1424	0.6019	0.4703	0.3369	0.6014	0.3100	1.0000	
SVI_t-1_	0.2231	0.5487	0.5270	0.5088	0.8795	0.4301	0.7047	0.6813	0.5702	1.0000

**Table 2:** The correlation coefficients between the realized volatility and the proxy variables.

	INR_t_	SMT_t_	TURN_t_	FUND_t_	SVI_t_	INR_t-1_	SMT_t-1_	TURN_t-1_	FUND_t-1_	SVI_t-1_
RV_SSECI_	0.2671	0.4023	0.5565	0.1910	0.7769	0.4120	0.5195	0.6417	0.1583	0.6814
RV_SZSME_	0.2424	0.3057	0.5817	0.1580	0.7074	0.3170	0.4062	0.6219	0.0846	0.6429
RV_CHINEXT_	0.2534	0.3616	0.5520	0.1980	0.7657	0.3670	0.4847	0.6109	0.1690	0.6848

**Table 3:** Results of the first step PCA.

	Percent variance explained (%)	Cumulative percent explained (%)		Percent variance explained (%)	Cumulative percent explained (%)
1	67.30%	67.30%	5	3.76%	96.23%
2	12.30%	79.60%	6	2.60%	98.83%
3	6.88%	86.48%	7	1.03%	99.86%
4	5.99%	92.47%	8	0.14%	100.00%

Then, the first principal component analysis is implemented.

From Table 3, we can see that the explanatory power of the first principal component is 68.87% and the cumulative explanatory power of the first three principal components is 85.20%. We choose the first component as a temporary index. Then we use the correlation between each variable and the first component to determine whether the current variable or the lagging one should be kept in the second step. In this way, the lead-lag relationships between different proxy variables are clearer and we can also retain the crucial information from a small set of variables. The correlation coefficients between the first component and each of the current or lagging variables are shown in Table 4. The variable and its lagging value are taken as a pair and the one with stronger correlation in each pair is selected for the second step PCA.

**Table 4:** Correlation coefficients between proxy variables and the first component.

Correlation coefficient with first component	INR_t_	SMT_t_	TURN_t_	SVI_t_
	0.6431	0.8974	0.8006	**0.9090**
Correlation coefficient with first component	INR_t-1_	SMT_t-1_	TURN_t-1_	SVI_t-1_
	**0.7456**	**0.9265**	**0.8298**	0.7748

From Table 4, we can see that SVI_t_ is more closely related to the temporary index than its lagging value. While the correlation coefficients between INR_t-1_, SMT_t-1_, TURN_t-1_ and the temporary index are higher than those of INR_t_, SMT_t_, and TURN_t_. INR_t-1_ is chosen instead of INR_t_ because Chinese people are prudent and risk-averse, e.g., they prefer savings rather than credit consumption. Investors prefer to practice with a small amount of money for a short period. Thus, the lag value of the number of new stock accounts is more closely related to investor sentiment. Financing transactions and short selling are two signals of market trends. As a result, margin balance, i.e., the integration of the financing and short-sell balances, can lead the market. With regard to TURN, the selection is the same as that of Baker and Wurgler [17].

Therefore, this paper chooses INR_t-1_, SMT_t-1_, TURN_t-1_ and SVI_t_ to constitute the final proxy variable set. We use PCA in a second step to obtain the cumulative percent explained by the components (Table 5).

**Table 5:** Results of the second step PCA.

	Eigenvalues	Percent variance explained (%)	Cumulative percent explained (%)
1	3.0413	75.56	75.56
2	0.5869	14.58	90.14
3	0.2808	6.97	097.11
4	0.1162	2.89	100.00

As can be seen from Table 5, the percentage variance explained by the first principal component is 75.56%, which is much higher than that in Baker and Wurgler [17]. Thus, we conclude that the first factor captures much of the common variation. The resulting index is

$$SENTIMENT_{t}=0.5341INR_{t-1}+0.4391SMT_{t-1}+0.5319TURN_{t-1}+0.4889SVI_{t}$$ (1)

**Fig 3:**
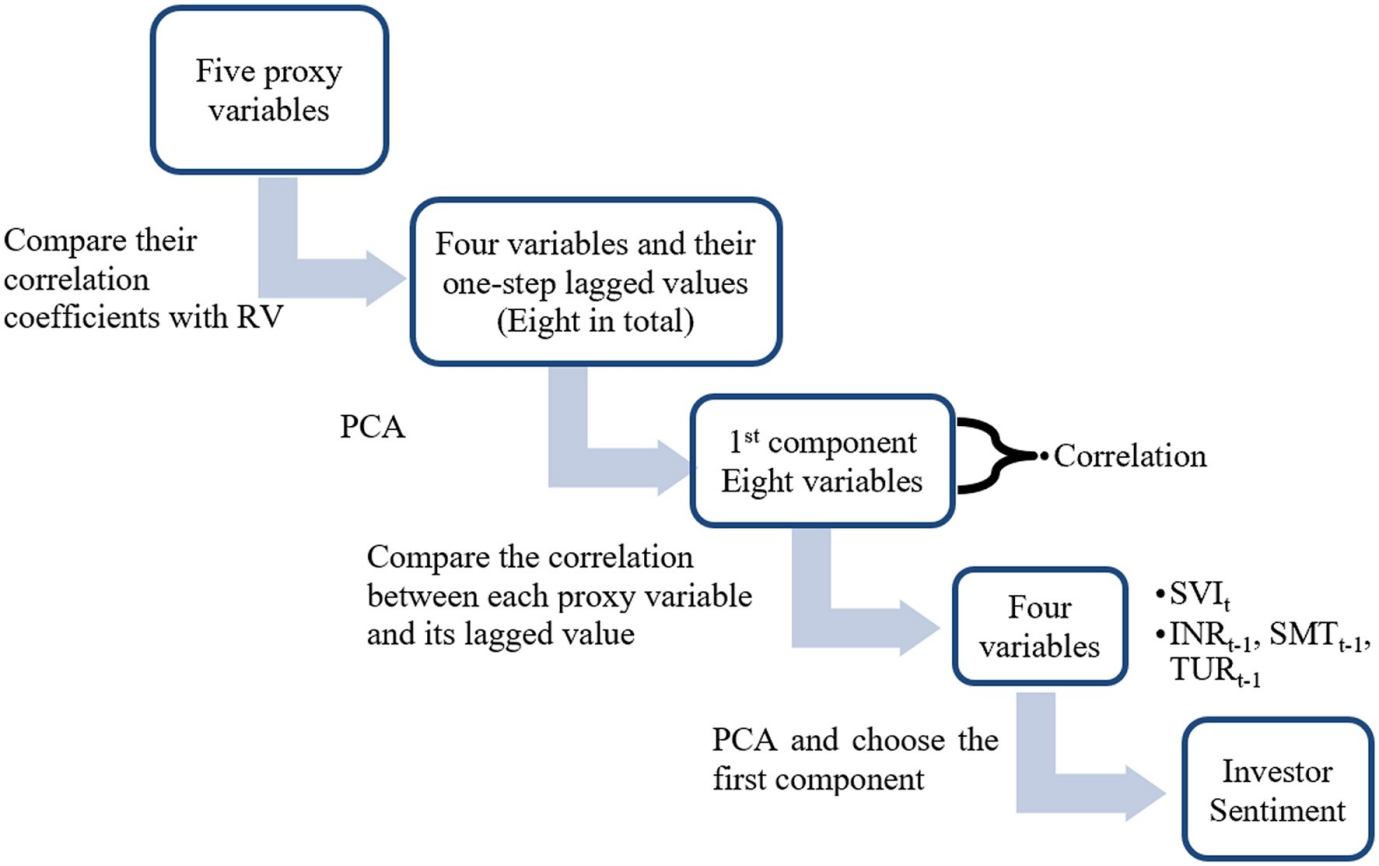
Process of constructing investor sentiment.

In the end of this section, we provide a sketch to illustrate the procedure of the two-step PCA used in this paper (Fig 3).

## Investor sentiment and realized volatility: Dynamic lead-lag analysis

### Thermal optimal path

The TOP method, which was originally called optimal thermal causal path, is a nonparametric method proposed by Sornette and Zhou [33] for identifying and quantifying the time-varying lead–lag structure between two time series. In a complex system, the lead-lag relationship between two time series will not stay the same. As a result, the dynamic measurement of this relation is crucial. From this dynamic measurement, the gradual changes in the lead-lag structure can be observed and can provide explicit guidance for researchers. Sornette and Zhou [33] also listed other advantages of TOP over similar traditional econometric methods. In the past decade, many studies have used the TOP method to analyze the lead-lag relationship between two time series associated with economic and financial cases [7, 34–37]. The key idea of this method is not complicated and includes just two steps which can be summarized as follows:

1. Define the distance matrix *E*_*X*,*Y*_ between two standardized time series *X* and *Y*. The elements in *E*_*X*,*Y*_ are calculated as:
  $$\varepsilon (t_{1},t_{2})=|X(t_{1})-Y(t_{2})|$$ (2)
  In this paper, *X* and *Y* have the same length, i.e., *t*_*1*_, *t*_*2*_ = *1*,*2*,*3*,*…*,*N*. The *N* × *N* matrix *E*_*X*,*Y*_ thus embodies all possible point-wise pairwise comparisons between the two time series.
2. Determine an optimal path that quantifies the lead-lag dependence between the two time series. The optimal path can be searched for in the form of a one-to-one mapping *t*_2_ = *ϕ*(*t*_1_) to make *X*(*t*_1_) and *Y*(*ϕ*(*t*_1_)) match best. In other words, we would like to determine a sequence of elements of the above distance matrix along which the elements are the smallest.

Although the idea is intuitive, a kind of smoothness requirement, equivalent in most cases to the continuity and monotonicity of the path, means that we cannot directly find the minimum value for each row of matrix *E*_*X*,*Y*_. The optimal path for two identical time series is the main diagonal; thus, the optimal path for two different but correlated time series is expected to wander around the main diagonal. It is convenient to use the rotated coordinate system (*x*, *t*) such that:

$$\{\begin{array}{l} x=t_{2}-t_{1}\\ t=t_{2}+t_{1} \end{array}$$ (3)

where *t* is in the main diagonal direction of the (*t*2, *t*1) system and *x* is perpendicular to *t*. Then, the path with *x*(*t*) = 0 defines synchronous patterns, while paths with *x*(*t*) ≠ 0 define varying lead–lag patterns. A positive *x* corresponds to *t*2 *> t*1 and means that the second time series Y(*t*_*2*_) lags behind the first time series *X*(*t*_*1*_), and vice versa.

To obtain the TOP trajectory ⟨*x(t)*⟩, partition functions *G(x*, *t)* for all values of *x* at a fixed *t* in the lattice are first calculated. Thus, *G(x*, *t)/G(t)* can be interpreted as the probability for a path to be at distance *x* from the diagonal for a distance *t* along the diagonal. This probability is determined as a compromise between minimizing the mismatch or cost as defined above (similar to an “energy”) and maximizing the combinatorial weight of the number of paths with similar mismatches in a neighborhood (similar to an ‘‘entropy”). Therefore, the recursive equation on *G(x*, *t)* is:

$$G_{⊲}(x,t)=[G_{⊲}(x-1,t-1)+G_{⊲}(x+1,t-1)+G_{⊲}(x,t-2)]e^{-\varepsilon (x,t)/T}$$ (4)

where the parameter *T* plays the role of a ‘‘temperature” controlling the relative importance of cost versus combinatorial entropy. A large value for *T* will wash out too much of the relevant signals, while a small value for *T* will extract too much from the spurious signals. Then, the TOP 〈*x*(*t*)〉 is obtained by the following formula:

$$\langle x(t)\rangle =\sum_{x}xG_{⊲}(x,t)/G_{⊲}(t)$$ (5)

### Empirical results

The stock market’s monthly realized volatility should be measured first. In this papers we measure the monthly realized volatility with the squared root of the aggregated squared returns. Following Siriopoulos and Fassas [38], we compute the return squared without mean-reversion assumption. We subtract the risk-free rate from the returns according to capital asset pricing models proposed by William Sharp because we are more concerned on the volatility of abnormal returns. In particular, the monthly realized volatility is calculated according to the following equation:

$$RV_{m}=\sqrt{\sum_{t=1}^{N_{m}}{\left(r_{t}-r_{riskfree}\right)}^{2}}$$ (6)

where, *r*_*t*_ is the daily return of stock index on day *t*, *N*_*m*_ is the number of trading days in month *m* and *r*_*riskfree*_ is the risk-free rate. In this paper, we use the overnight Shanghai Interbank Offered Rate as the risk-free rate.

When checking the lead-lag relationship, the investor sentiment is taken as the first time series and the stock market realized volatility is taken as the second series. As for the choice of the temperature *T*, based on earlier literature on the TOP method [7], the result is robust when *T* = 3 and qualitatively similar results are obtained with respect to variations of *T* between 1 and 3.

Three different stock indices, which represent three different levels in the Chinese stock market, are included in this research. The SSECI is a stock market index of all stocks that are traded at the SSE. Most of the listed enterprises in the SSE are state-owned and well-capitalized, such as the Industrial and Commercial Bank of China, China Unicom and China Eastern Airlines. The SSECI represents the major power of the Chinese stock market. The second index is the SZSE’s Small and Medium Enterprise Price index (SZSME), which represents the economic situation of Chinese small and medium enterprises(SMEs). The final index is the Chinese Growth Enterprise Index or ChiNext Price Index, which represents the power of entrepreneurial enterprises and high-tech enterprises that cannot be listed on the main board. However, the Shenzhen Component Index is excluded from this research. Although it is the second most important stock index in China, it is calculated based on the prices of stocks listed in the main board, SME board, and growth enterprise market (GEM). Thus, it cannot reflect the main, SME, or GEM boards singly. Table 6 displays the basic descriptive statistics for the three indices’ realized volatility. The ChiNext Price Index is the most volatile one because the enterprises in this section are not capitalized well and even a large investor can pump and dump. In the opposite, SSECI has the lowest volatility. Abundant capital makes the stock price can’t be manipulated easily.

**Table 6:** Descriptive statistics of realized volatilities.

Statistic	SSECI	SZSME	CHINEXT
Mean	0.0545	0.0695	0.0824
Max	0.1781	0.1828	0.2122
Min	0.0126	0.0252	0.0287
Median	0.0438	0.0602	0.0718

**Fig 4:**
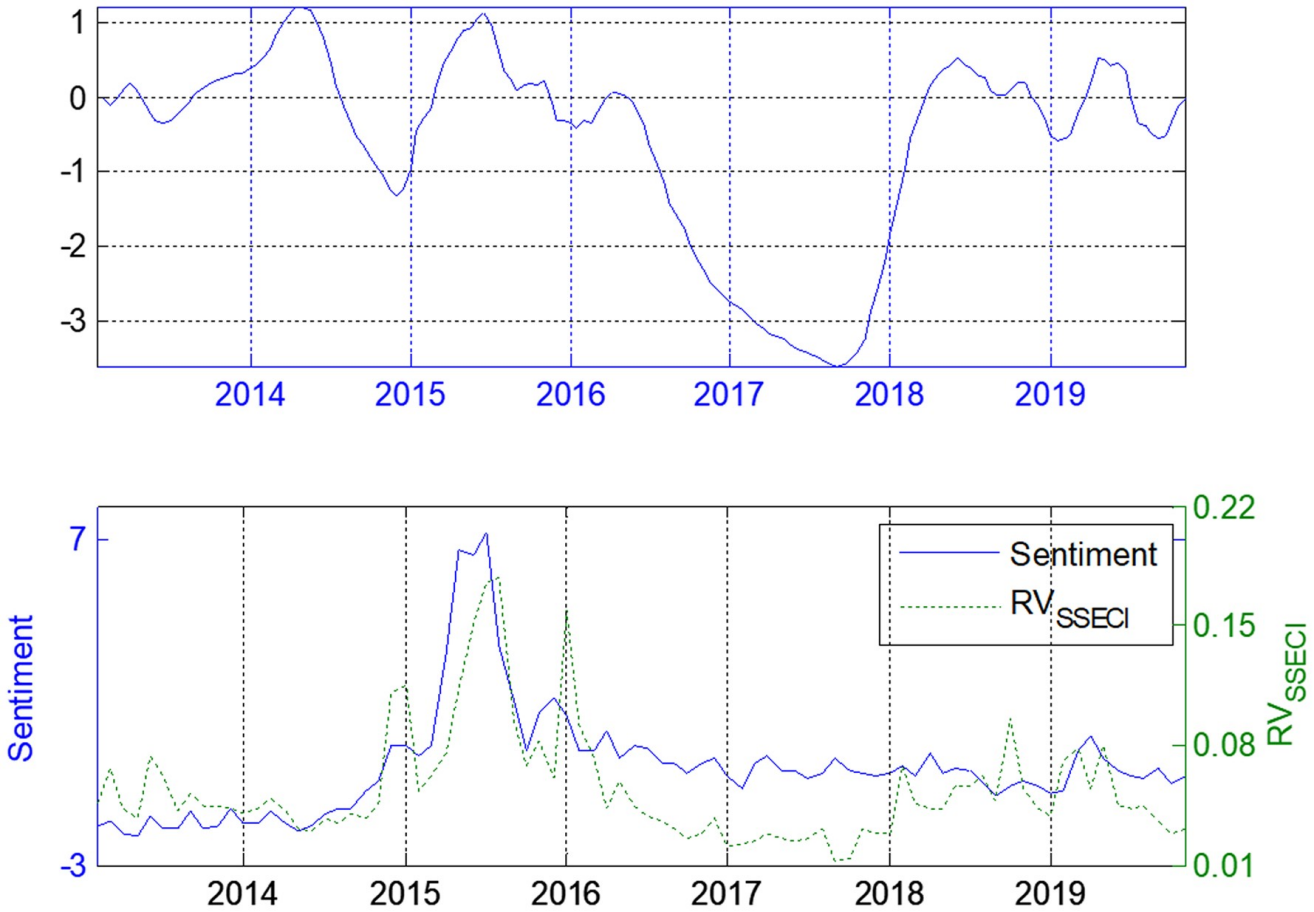
Lead-lag structure for investor sentiment and SSE Composite Index.

**Fig 5:**
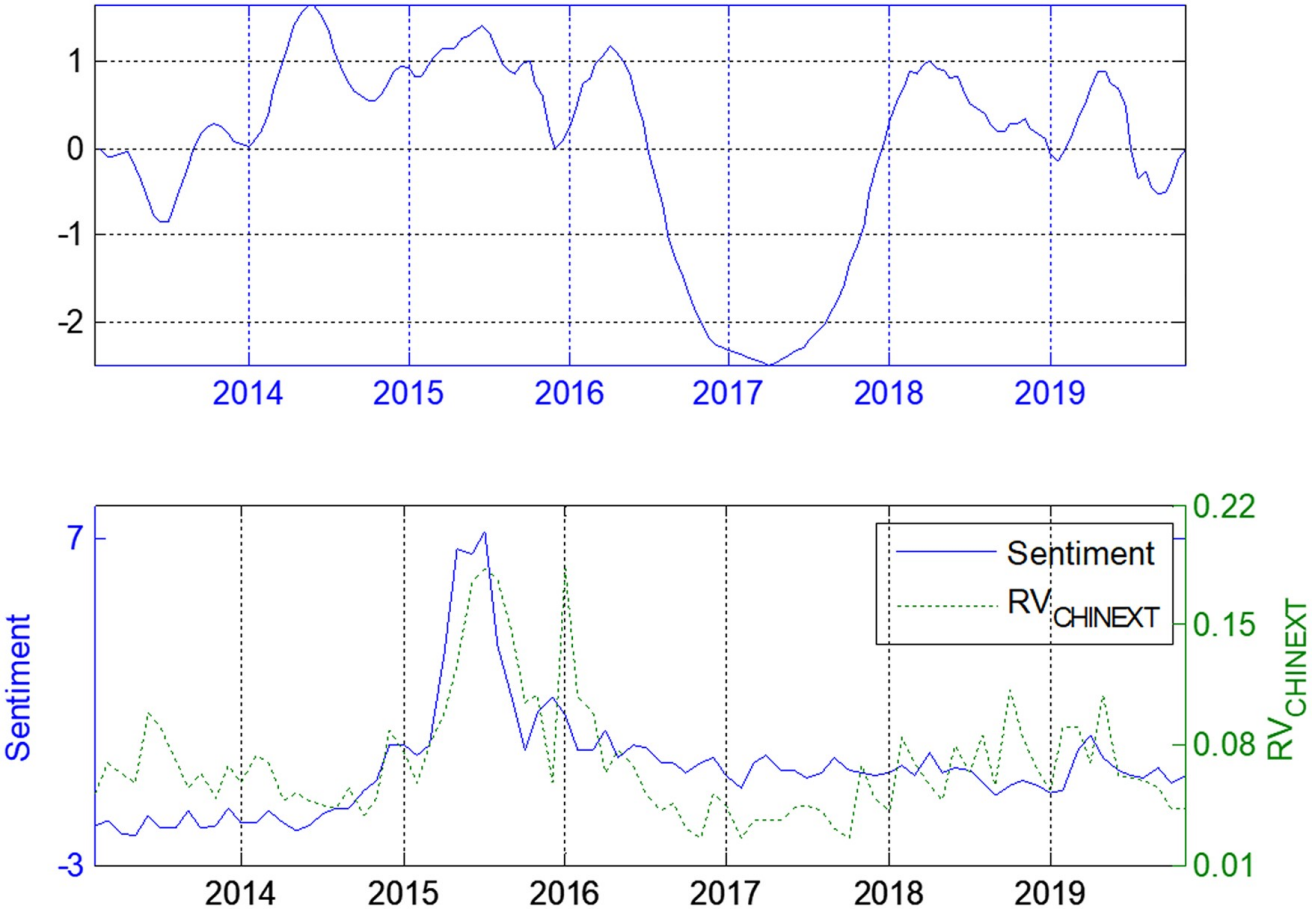
Lead-lag structure for investor sentiment and ChinNext Price Index.

**Fig 6:**
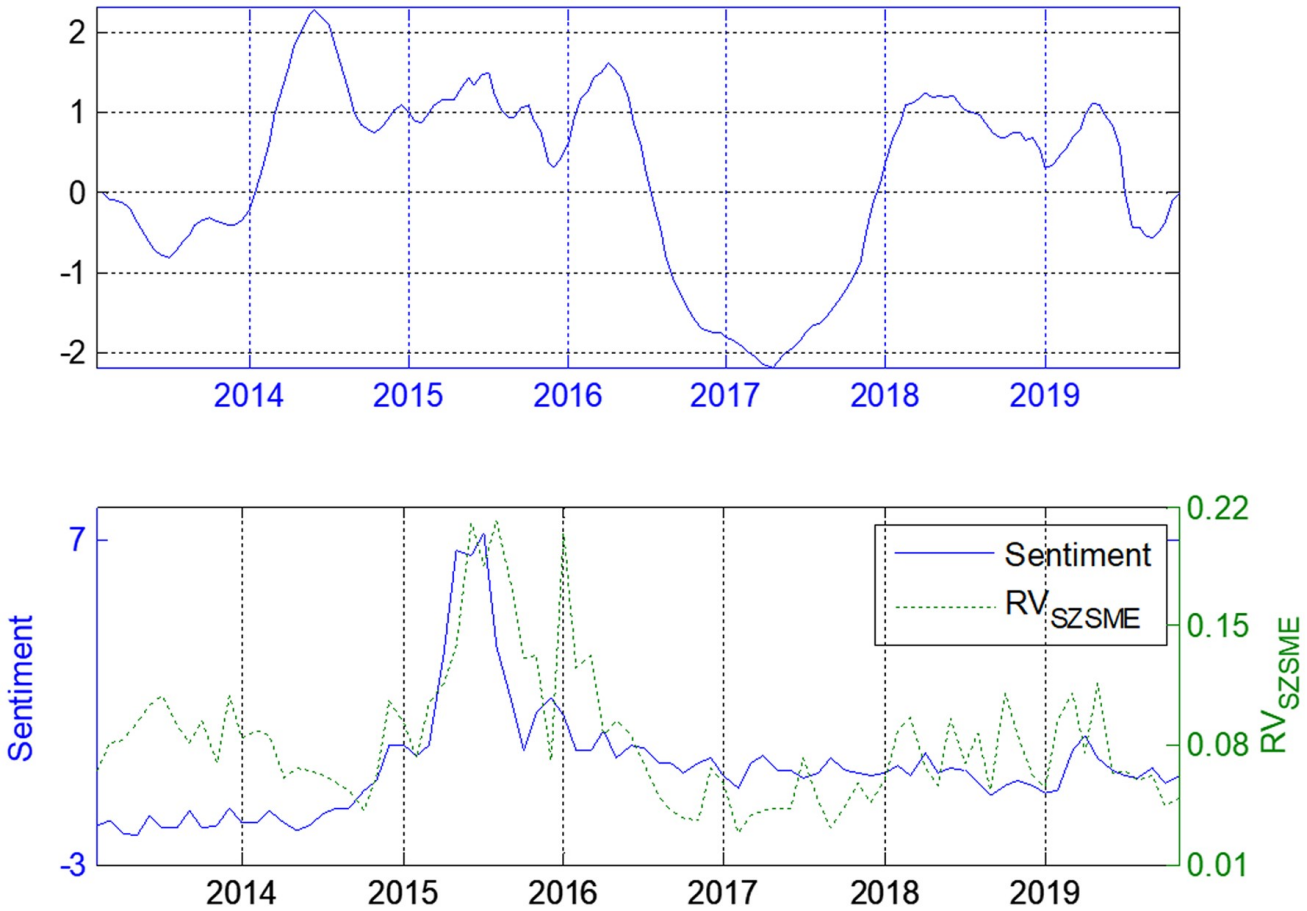
Lead-lag structure for investor sentiment and SZSME Price Index.

Figs 4–6 show the dependence of the lead–lag *x(t)* between the investor sentiment and the realized volatilities of SSE Composite, SZSME and ChiNext Price Index respectively. The unit of *x(t)* is one month. The investor sentiment does not always lead the stock market since *x(t)* is not always positive. When there is less fluctuant in the market, the investor sentiment almost always lags behind the stock market (e.g. see the period from last half of 2016 to the end of 2017). When the investors are enthusiastic, such as during the soaring and the shock in 2015, investor sentiment leads the realized volatility for about one step on average in all three markets. This is also true during the period from January 2018 to the middle of 2019.

## Discussion

In this section, we discuss whether the use of a sentiment lagged by one order can dominate single proxy variable in reducing the proportion of total variation in modeling the realized volatility with a multivariate autoregressive moving average (ARMA(1,1)). As an ARMA model requires the variables to be stationary, we check whether the variable is stationary through three different tests. The results are shown in S2 Table. According to three tests, we can conclude that the variables are approximately stationary and the ARMA model is applicable here. The predictive power of our sentiment index is also checked in this section. ARMA is the most widely recognized basic way to predict economic and financial data and it can be included in complex models. This paper takes investor sentiment or a single proxy variable as the exogenous variable in ARMA and compares the coefficients of determination, because the coefficient of determination (R^2^) is interpreted as the proportionate reduction of total variation associated with the use of the exogenous variable. In Table 7, the investor sentiment is calculated by the number of new stock accounts, margin balance, turnover ratio and investor attention. As can be seen in Table 7, all coefficients are significantly different from zero. *SENTIMENT*_*t-1*_ impacts realized volatility positively. When the model includes *SENTIMENT*_*t-1*_, the coefficients of determination increase by 25% averagely.

**Table 7:** Estimated parameters of ARMA(1,1) with and without investor sentiment index.

	RV_SSECI_		RV_SZSME_		RV_CHINEXT_	
constant	0.0535	0.0556	0.0674	0.0705	0.0795	0.0833
	(0.0011)	(0.0000)	(0.0002)	(0.0000)	(0.0002)	(0.0000)
AR(1)	0.7487	0.9215	0.8508	0.9165	0.8732	0.9297
	(0.0000)	(0.0000)	(0.0000)	(0.0000)	(0.0000)	(0.0000)
MA(1)	-0.0739	-0.6278	-0.3140	-0.6207	-0.3490	-0.6035
	(0.6223)	(0.0001)	(0.0046)	(0.0002)	(0.0003)	(0.0000)
SENTIMENT_t-1_		0.0148		0.0138		0.0148
		(0.0000)		(0.0001)		(0.0000)
R^2^	0.5183	0.6743	0.5232	0.6577	0.5493	0.6681
Adjusted R^2^	0.4998	0.6571	0.5049	0.6397	0.5319	0.6507
F-statistic	27.9805	39.3304	28.5329	35.5081	31.6860	38.2543

Note: The number in the bracket is the probability of the parameters t-statistics.

If one single proxy variable is taken as the exogenous variable in the ARMA model, the parameters are estimated and shown in Table 8. Table 8 also presents the estimated results if the investor sentiment is calculated without considering investor attention. Not all proxy variables are significantly different from zero in the models. The parameters of the number of new stock accounts (INR_t-1_) are nearly zero when estimating three realized volatilities. In Table 8 all the coefficients of determination are smaller than that of the model which uses investor sentiment as the exogenous variable in Table 7. Thus, we can conclude that the investor sentiment index that considers search volume reduces the total variation.

**Table 8:** Estimated parameters in ARMA(1,1) with different exogenous variables.

Model Specification		constant	AR(1)	MA(1)	PV_t-1_	R^2^	Adjusted R^2^	F-statistic
ARMA(1,1)+INR	RV_SSECI_	0.0518***	0.7342***	-0.0747	1.78E-05	0.5256	0.5006	21.0494
	RV_SZSME_	0.0669***	0.8512***	-0.3314***	1.06E-05	0.526	0.501	21.083
	RV_CHINEXT_	0.0809***	0.8740***	-0.3465***	-3.73E-06	0.551	0.5274	23.3162
ARMA(1,1)+SMT	RV_SSECI_	0.0068	0.8609***	-0.3859***	6.38E-06***	0.5882	0.5665	27.1363
	RV_SZSME_	0.0263	0.8931***	-0.4621***	5.78E-06***	0.588	0.5663	27.1191
	RV_CHINEXT_	0.0434**	0.9040***	-0.4647***	5.14E-06***	0.588	0.5663	27.1121
ARMA(1,1)+TURN	RV_SSECI_	0.0355**	0.7370***	-0.2004	0.0066**	0.5347	0.5102	21.8308
	RV_SZSME_	0.0508**	0.8472***	-0.4081***	0.0063**	0.539	0.5148	22.2183
	RV_CHINEXT_	0.0608***	0.8723***	-0.4413***	0.0072**	0.5659	0.5431	24.7714
ARMA(1,1)+SVI	RV_SSECI_	0.0327***	0.8779***	-0.5858***	2.22E-07***	0.6051	0.5843	29.1133
	RV_SZSME_	0.0489***	0.8896***	-0.6114***	2.1E-07***	0.601	0.5800	28.6137
	RV_CHINEXT_	0.0609***	0.9116***	-0.6002***	2.15E-07***	0.6122	0.5918	29.9960
ARMA(1,1)+Investor sentiment without SVI	RV_SSECI_	0.0544***	0.8517***	-0.5041***	0.0109***	0.5744	0.5520	25.6405
	RV_SZSME_	0.0692***	0.8712***	-0.5035***	0.0100**	0.5836	0.5617	26.6271
	RV_CHINEXT_	0.0818***	0.8899***	-0.4943***	0.01093***	0.6092	0.5886	29.6162

Note:

*, ** and *** indicate, respectively, the cumulative abnormal return is significantly different from zero at the 10%, 5% and 1% levels.

Considering predictive power comparison, this research uses one-step-ahead prediction and takes the last seven observations (from May 2019 to November 2019) as the out-of-sample data. Root Mean Square Error (RMSE) and Theil’s inequality coefficient are used to evaluate the prediction performance. RMSE refers to the prediction errors. Theil’s inequality coefficient provides a measure of how well the predicted values compares to the observed values. The smaller the RMSE and Theil’s inequality coefficient are, the better the model performs. As shown in Table 9, investor sentiment which is constructed with INR, SMT, TURN and SVI (noted as SENTIMENT in Table 9) helps ARMA(1,1) achieve the smallest RMSE and Theil’s inequality coefficient. Investor attention also improves the prediction accuracy, but it performs a little worse than the investor sentiment constructed with INR, SMT, TURN and SVI. Investor sentiment index constructed with INR, SMT, TURN ranks third in the prediction accuracy. Thus, it can be concluded that investor sentiment can forecast realized volatility. If investor attention is considered in constructing the investor sentiment, the prediction accuracy will be improved further.

**Table 9:** The out-of-sample prediction performances of different model specifications.

	RV_SSECI_		RV_SZSME_		RV_CHINEXT_	
	RMSE	Theil’s inequality	RMSE	Theil’s inequality	RMSE	Theil’s inequality
ARMA(1,1)	0.0159	0.1640	0.0197	0.1466	0.0217	0.1496
ARMA(1,1)+SENTIMENT	**0.01334**	**0.1312**	**0.0159**	**0.1157**	**0.0185**	**0.1248**
ARMA(1,1)+Investor sentiment without SVI	0.0149	0.1512	0.0188	0.1394	0.0207	0.1420
ARMA(1,1)+INR	0.0158	0.1631	0.0196	0.1456	0.0218	0.1502
ARMA(1,1)+SMT	0.0154	0.1527	0.0194	0.1412	0.0217	0.1466
ARMA(1,1)+TURN	0.0168	0.1753	0.0204	0.1536	0.0221	0.1535
ARMA(1,1)+SVI	0.0147	0.1464	0.0176	0.1297	0.0201	0.1365

## Concluding remarks

In this paper, we first summarize the three primary methods of measuring investor sentiment in previous researches. After analyzing their pros and cons, we follow Baker and Wurgler’s method and construct an investor sentiment based on four proxy variables selected according to the real-world situation in the Chinese stock market. Three of the proxy variables are statistics data associated with the stock market, one of the proxy variables is the searching volume index, which was a recent concern. Using a two-step PCA to retain the most information of the proxy variables, we construct an investor sentiment. Few researchers have focused on the dynamic lead-lag relationship between investor sentiment and realized volatility; therefore, we use TOP, a method obtained from the complex system theory, to analyze the time-varying characteristics of the relationship between investor sentiment and stock market realized volatility. We find that when investor sentiment rockets, it indicates the market will experience a shock in the following month. This is significant information, which can be used by the government and related institutions to prevent financial risk in China. This paper then discusses the variation reduction effect of the sentiment index based on ARMA(1,1). The results show that when taking search volume index as a proxy variable to calculate the investor sentiment index, the investor sentiment index accounts for more variation reduction in estimating future realized volatility. Finally, the prediction power of our investor sentiment index is checked using RMSE and Theil’s inequality coefficient. The results indicate the investor sentiment index considering search volume index performs better than taking other variables as exogenous variables in ARMA(1,1). In the next step, we will continue our research in two directions within the Chinese stock market: the first is to use higher frequency statistical data to measure the investor sentiment, while the second is to combine complex system theory with a traditional econometric method to explore the complicity between investor sentiment and market volatility.

## Supporting information

S1 Table Daily average search volume of the potential keywords (from April 1^st^, 2012 to November 30^th^, 2019). (DOCX)

S2 Table The unit root test. (DOCX)

S1 Data (XLSX)
