# Supplementary material for: A dynamic analysis of the relationship between investor sentiment and stock market realized volatility: Evidence from China
Source: PLoS One. 2020 Dec 4;15(12):e0243080. doi: 10.1371/journal.pone.0243080 (PMC7717912; doi:10.1371/journal.pone.0243080)
Supplement: S1 Table — (DOCX) [file pone.0243080.s001.docx]

S1 Table. Daily Average Search Volume of the potential Keywords (from April 1^st^, 2012 to November 30^th^, 2019 )

| **No.** | **Keyword in Chinese phonetic alphabet** | Explanation in English | Daily Average search Volume^a^ |
| --- | --- | --- | --- |
| 1 | Shang Zheng Zhi Shu | Stock Index of Shanghai Stock Exchange | 90098 |
| 2 | Shen Zheng Zhi Shu | Stock Index of Shenzhen Stock Exchange | 1848 |
| 3 | Shen Cheng Zhi | An abbreviation of Shenzhen component index | 331 |
| 4 | Shen Zheng Cheng Zhi | An abbreviation of Shenzhen component index | 2626 |
| 5 | Shang Zheng Zong Zhi | An abbreviation of Shanghai composite index | 2601 |
| 6 | Shang Zheng Zong He Zhi Shu | Shanghai composite index | 365 |
| 7 | Shen Zheng Chen Fen Zhi Shu | Shenzhen Component Index | *Not included in Baidu Index* |
| 8 | Gu Shi | Stock Market | 12994 |
| 9 | Gu Shi Hang Qing | Stock Market quotation | 16588 |
| 10 | Gu Piao | Stock | 22915 |
| 11 | Gu Piao Hang Qing | Stock quotation | 16784 |
| 12 | Gu Piao Zhi Shu | Stock Index | 528 |
| 13 | Jin Ri Da Pan | Today’s main board quotation | 1590 |

^a^The Daily Average search Volumes are provided by <http://index.baidu.com>.
