# Supplementary material for: A dynamic analysis of the relationship between investor sentiment and stock market realized volatility: Evidence from China
Source: PLoS One. 2020 Dec 4;15(12):e0243080. doi: 10.1371/journal.pone.0243080 (PMC7717912; doi:10.1371/journal.pone.0243080)
Supplement: S2 Table — (DOCX) [file pone.0243080.s002.docx]

S2 Table. The Unit Root Test

| Unit Root Test | SSECI | SZSME | CHINEXT | SENTIMENT |  |
| --- | --- | --- | --- | --- | --- |
| ADF test | -3.5570^***a^ | -2.5201 | -2.2497 | -2.8427^*^ |  |
| PP test | -3.4581^***^ | -3.5281^***^ | -3.4189^***^ | -2.2760 |  |
| KPSS test | 0.1676 | 0.1251 | 0.2201 | 0.1817 |  |
|  | Investor sentiment without SVI | INR | SMT | TURN | SVI |
| ADF test | -2.8134^*^ | -5.2724^***^ | -2.9767^**^ | -3.9126^***^ | -3.0624^**^ |
| PP test | -2.8760^*^ | -5.2233^***^ | -2.3537 | -3.7753^***^ | -2.6098^*^ |
| KPSS test | 0.1695 | 0.1336 | 0.3194 | 0.2177 | 0.1703 |

^a^ ^*^, ^**^ and ^***^ indicate, respectively, the cumulative abnormal return is significantly different from zero at the 10%, 5% and 1% levels.
